# Hospital Meetings

**Published:** 1904-07-23

**Authors:** 


### HOSPITAL MEETINGS.

### ASSOCIATION FOR THE ORAL INSTRUCTION OF THE DEAF AND DUMB.

THE annual meeting of this Association was held on Tuesday, July 19th, the Rev. C. H. Parez presiding. Specimens of the pupils' handiwork were on view, and prizes to successful competitors were distributed by Miss Amy Paget, who received a basket of roses from a little scholar.

About 40 or 50 children are now under instruction and during the afternoon short illustrations of the system were given in the elementary, intermediate, and advanced stages.

The Chairman, in proposing the adoption of the annual report, said the institution continued to afford training in language and speech of a very high grade, as shown in the report of H.M.'s Inspector of Schools for 1904. The children who reached the highest classes showed a remarkable intelligence and facility with the common facts of life and the occurrences of the day, and they owed this and their happy disposition to the excellent influence and patient hard work of the teachers. The grant earned was again the highest, *i.e.*, £150 1s. 3d. Funds were urgently required, not only for maintaining the school, but for building a new workshop and gymnasium, while there was still an annual excess of expenditure over income of about £600. Part of the funds now invested would have to be sold in order to meet current expenses, unless many new subscriptions and donations were obtained.

Dr. Humphreys having seconded the motion, the report was adopted.

Mr. Van Praag, director, drew attention to the fact that while the demand for qualified teachers continued to increase, there were not sufficient applications for training students to meet it. He pointed out that the work of teaching the deaf offered to those disposed to engage in it an admirable and remunerative professional opening. The suggestion to delete the word "dumb" from the name of the association was under consideration.

